# Identification of the Regulatory Genes of UV-B-Induced Anthocyanin Biosynthesis in Pepper Fruit

**DOI:** 10.3390/ijms23041960

**Published:** 2022-02-10

**Authors:** Yihao Wang, Sujun Liu, Haoran Wang, Yingxue Zhang, Wenjie Li, Jinkui Liu, Qing Cheng, Liang Sun, Huolin Shen

**Affiliations:** 1 Beijing Key Laboratory of Growth and Developmental Regulation for Protected Vegetable Crops, China Agricultural University, Beijing 100193, China; niaoniao96@cau.edu.cn (Y.W.); liusujun213@163.com (S.L.); 18813013760@163.com (H.W.); b20203170837@cau.edu.cn (Y.Z.); wenjieli2290@163.com (W.L.); 18435709400@163.com (J.L.); chengqing2020@cau.edu.cn (Q.C.); 2 Department of Vegetable Science, College of Horticulture, China Agricultural University, No. 2 Yuanmingyuan Xi Lu, Haidian District, Beijing 100193, China; 3 Sanya Institute, China Agricultural University, Science and Technology City, Yazhou Bay, Sanya 572025, China

**Keywords:** pepper, anthocyanin biosynthesis, UV-B, transcription factor, Y1H, Y2H, VIGS

## Abstract

Fruit peels of certain pepper (*Capsicum annum* L.) varieties accumulate a large amount of anthocyanins and exhibit purple color under medium-wave ultraviolet (UV-B) conditions, which severely impacts the commodity value of peppers. However, the regulatory mechanism of the above process has not been well studied so far. To explore which key genes are involved in this regulatory mechanism, pepper variety 19Q6100, the fruit peels of which turn purple under UV-B conditions, was investigated in this study. Transcription factors with expression levels significantly impacted by UV-B were identified by RNA-seq. Those genes may be involved in the regulation of UV-B-induced anthocyanin biosynthesis. Yeast one-hybrid results revealed that seven transcription factors, CabHLH143, CaMYB113, CabHLH137, CaMYBG, CaWRKY41, CaWRKY44 and CaWRKY53 directly bound to the putative promotor regions of the structural genes in the anthocyanin biosynthesis pathway. CaMYB113 was found to interact with CabHLH143 and CaHY5 by yeast two-hybrid assay, and those three genes may participate collaboratively in UV-B-induced anthocyanin biosynthesis in pepper fruit. Virus-induced gene silencing (VIGS) indicated that fruit peels of CaMYB113-silenced plants were unable to turn purple under UV-B conditions. These findings could deepen our understanding of UV-B-induced anthocyanin biosynthesis in pepper.

## 1. Introduction

Anthocyanins are important secondary metabolites in plants and participate in the coloration of leaves, corolla and fruit [1]. They also play diverse biological roles in the life cycle of plants. For example, anthocyanins could protect plants from the harm of plenty of abiotic stresses, such as ultraviolet (UV) radiation, low temperature and drought [2,3,4]. Additionally, they can also attract insects and animals to pollinate and spread seeds [5,6].

The anthocyanin biosynthesis pathway, a branch of the flavonoid pathway, has been well studied previously, and most of the gene-encoding enzymes in the key step of anthocyanin biosynthesis have been investigated in many plant species [7,8]. Flavonoid biosynthesis starts with phenylalanine ammonia lyase (PAL) and 4-coumaryl:CoA ligase (4CL). Anthocyanin early biosynthetic genes (EBGs), including *Chalcone Synthase* (*CHS*), *Chalcone Isomerase* (*CHI*) and *Flavanone 3-Hydroxylase* (*F3H*), are also involved in the common flavonoid biosynthesis pathway. Anthocyanin late biosynthetic genes (LBGs), including *Flavonoid 3’5’-Hydroxylase* (*F3’5’H*), *Dihydroflavonol4-Reductase* (*DFR*), *Anthocyanidin Synthase* (*ANS*) and *UDP-Glucose: Flavonoid3-O-Glucosyltransferase* (*UFGT*), are necessary for the accumulation of anthocyanins and some special flavonoids [9,10]. EBGs and LBGs are also called anthocyanin structural genes, most of which are regulated by MYB transcription factors (TFs), basic-helix-loop-helix (bHLH) TFs and WD-repeat proteins, which form an MYB/bHLH/WD40 (MBW) complex. This complex plays a central role in the regulation of anthocyanin biosynthesis [11,12,13]. The regulatory relationship between MBW complex and LBGs has been well studied in many plant species, such as petunia [14,15], eggplant [16] and nectarine [17]. In addition to the members of the MBW complex, some other types of TF have also been identified to control the biosynthesis of anthocyanin, such as NAC, WRKY and zinc finger protein [18,19,20].

The biosynthesis and accumulation of anthocyanins is often affected by external environmental signals, such as light, temperature and drought [21]. Light is the main energy source and information signal that regulates plant growth and development and is also one of the most important environmental factors, essential for anthocyanin biosynthesis in various organs of plants [9,22]. Different wavelengths of light have different effects on the accumulation of anthocyanins. Compared with visible light, UV light has more obvious and profound effects on anthocyanin biosynthesis [23]. For instance, peach fruit was able accumulate plenty of anthocyanins under UV-A and UV-B conditions [24]. Strawberries treated with UV-C significantly increased their antioxidant capacity and anthocyanin content [25]. In apples, pears and tomatoes, anthocyanin content in the peel of the fruit was significantly increased by UV-B radiation [26,27,28].

Higher plants have specific photoreceptors to receive light signals of different wavelengths. UV Resistance Locus 8 (UVR8) is one such photoreceptor, specifically receiving UV-B light signals [29]. The UVR8 photoreceptor forms a monomer UVR8 after receiving a UV-B signal and competitively binds to the substrate binding site of COP1, thereby inhibiting the activity of its E3 ubiquitin ligase on the target protein, such as HY5, regulating the expression of downstream transcription factors and anthocyanin structural genes to enhance the accumulation of anthocyanin [30,31,32]. HY5 not only plays an important role in the regulation of UV-B-induced anthocyanin biosynthesis but also participates in the regulation pathways of anthocyanin biosynthesis, which are induced by other light signals [26,33,34,35]. In Arabidopsis, HY5 binds directly to members of the MBW complex, mostly MYB TFs, such as AtMYB12 and AtMYB75, to promote their expression and regulate anthocyanin biosynthesis [36,37].

In pepper, silencing of *CaMYB* via virus-induced gene silencing (VIGS) led to the downregulation of the expression of structural genes and caused a decrease in anthocyanin accumulation [38,39]. *CaMYBA*, a gene that regulates anthocyanin biosynthesis in purple flowers, leaves, and immature fruits of pepper, is located on chromosome 10 [40]. CaANT1, CaANT2, CaAN1 and CaTTG1 participated in the accumulation of anthocyanin in pepper through formation of a new MMBW transcription complex [41]. However, most current research on anthocyanin biosynthesis in pepper mainly focuses on the materials that naturally bear purple fruit, with little focus on the study of light-induced purple fruit. Due to the above reasons, the regulatory mechanism of UV-B-induced anthocyanin biosynthesis is still unclear.

In this study, fruits of 19Q6100 were used as material, of which some part of the peel could accumulate anthocyanin when exposed to natural sunlight. Transcription factors that were significantly differentially expressed between purple and green peels were identified by RNA-seq. The interaction among CaMYB113, CabHLH143 and CaHY5, as well as the interaction between the abovementioned three TFs and anthocyanin structural genes, was verified by yeast one-hybrid (Y1H) and yeast two-hybrid (Y2H). The function of *CaMYB113* in UV-B-induced anthocyanin biosynthesis in pepper was discovered. Results of this work could deepen our understanding of the regulatory mechanism of anthocyanin biosynthesis in pepper and provide useful information for the future study of UV-B-induced anthocyanin accumulation in fruit peels of other species.

## 2. Results

### 2.1. Anthocyanin Content and Expression of Anthocyanin Biosynthesis Genes Were Induced by UV-B in the Peel of 19Q6100

**Figure 1:**
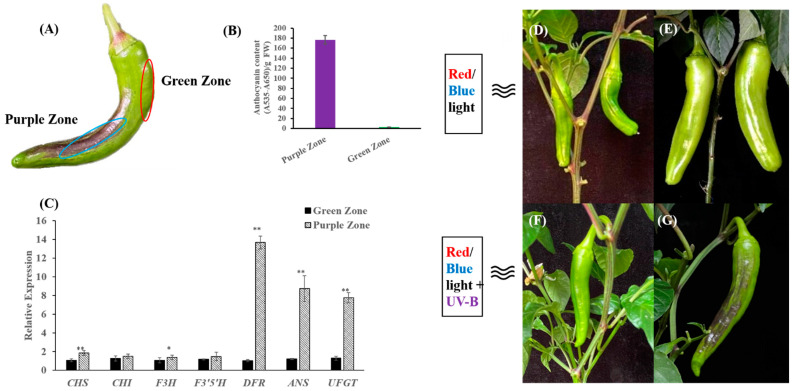
Analysis of anthocyanin contents, light response and expression of anthocyanin structural genes induced by UV-B in 19Q6100 pepper fruit. (**A**) Phenotype of UV-B-induced pepper fruit peels. (**B**) Anthocyanin contents in purple zone and green zone of pepper fruit peels after UV-B exposure. Numerical values are presented as means (± SE) from three independent biological replicates. (**C**) Relative expression of anthocyanin structural genes between purple zone and green zone of pepper fruit peels. Statistically significant differences between purple zone and green zone are indicated by Student’s *t*-test. (** *p* < 0.01, * *p* < 0.05). (**D**,**E**) Growth condition of pepper fruits before treatment and under red/blue light for 5 days, respectively. (**F**,**G**) Growth conditions of pepper fruits before treatment and under red/blue + UV-B light, respectively, for 5 days.

A natural pepper mutant of which the sun-facing side of the mature green fruit accumulates anthocyanin in the peel was discovered in our experimental field. A stable inbred line 19Q6100 was then generated via selfing. The sun-facing side of 19Q6100 fruit was purple, while the shaded side was still green (Figure 1A). In order to clarify what kind of light could induce the accumulation of anthocyanin in the peel, mature green fruits of 19Q6100 were grown under different wavelengths of light. The fruit peel under red/blue light for 5 days did not turn purple (Figure 1D,E), while the fruit peel treated with red/blue + UV-B for 5 days turned purple (Figure 1F,G), suggesting that UV-B light may be the key factor that induces the accumulation of anthocyanin in the peels of peppers. Later, anthocyanin content was measured in purple and green parts of the red/blue + UV-B-treated fruits. As shown in Figure 1B, the anthocyanin level was significantly higher in the purple part than that in the green part. For further confirmation of the accumulation of anthocyanin, the expression of genes encoding the key enzymes involved in anthocyanin biosynthesis was investigated. As expected, expression of all of the anthocyanin structural genes was significantly induced in the purple peels, except for *CHI* (Figure 1C). Therefore, it can be presumed that some of the upstream regulatory genes may be induced by UV-B light.

### 2.2. Transcriptome Analysis of Purple and Green Peels of UV-B-Treated Fruits

**Figure 2:**
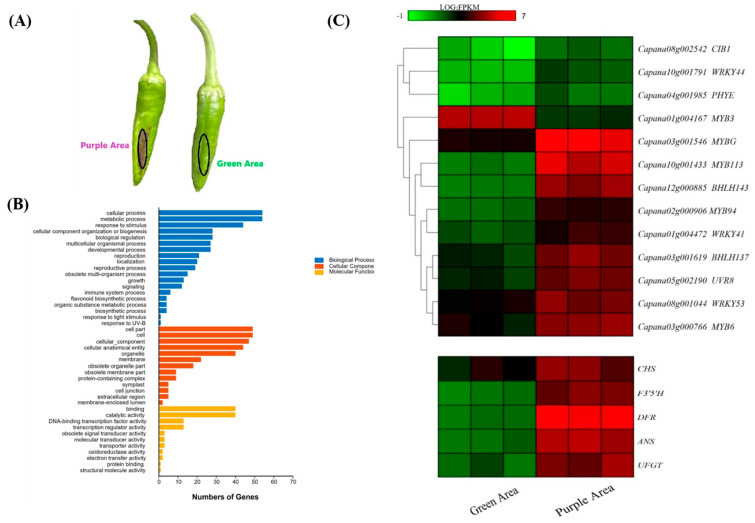
Transcriptome analysis between purple area and green area of pepper fruit. (**A**) Sampling site of pepper fruit for RNA-seq. (**B**) GO pathway analysis of differentially expressed genes. (**C**) FPKM analysis of anthocyanin-biosynthesis-related regulatory genes and anthocyanin structural genes among differentially expressed genes between purple area and green area. The color scale from green to red indicates the value of log_2_FPKM.

RNA-seq was performed to investigate the transcriptome changes caused by UV-B. The purple and green peels of the UV-B-treated fruits were sampled and sequenced (Figure 2A). The ratios of the mapped reads vary from 86.40% to 93.74% in all samples (Supplementary Table S1). The correlation coefficients (R2) between the biological replicates were 0.935 (Green peel-1 vs. Green peel-2), 0.932 (Green peel-1 vs. Green peel-3), 0.883 (Green peel-2 vs. Green peel-3), 0.922 (Purple peel-1 vs. Purple peel-2), 0.933 (Purple peel-1 vs. Purple peel-3) and 0.954 (Purple peel-2 vs. Purple peel-3) (Supplementary Table S2). Fragments per kilobase of transcript per million mapped reads (FPKMs) were calculated, and a total of 108 DEGs were identified between the purple and green peels, among which 71 and 37 genes were up- and downregulated, respectively, in the purple peel (Supplementary Table S3). Gene ontology (GO) analysis revealed that DEGs were significantly enriched in the “cellular process” and “metabolic process” with respect to biological process, “cell part” and “cell” with respect to cellular components and “binding” and “catalytic activity” with respect to molecular function (Figure 2B).

### 2.3. Transcription Factors and Structural Genes Involved in UV-B-Induced Anthocyanin Biosynthesis

To further illustrate the changes caused by UV-B at the transcription level, 9 transcription factors (TFs), 5 structural genes and 4 light responses were identified among the 108 DEGs (Figure 2C). Expression patterns of some of the abovementioned genes were further confirmed via qRT-PCR (Supplementary Figure S1). Based on the expression patterns, TFs and light responses could be roughly divided into three types: (I) expressed only in the purple parts of peels, including *CIB1*, *WRKY44* and *PHYE*; (II) expressed at higher levels in the purple peels, including *MYBG*, *MYB113*, *bHLH143*, *MYB94*, *WRKY41*, *bHLH137*, *UVR8*, *WRKY53* and *MYB6*; (III) expressed at higher levels in the green peel, such as *MYB3* (Figure 2C). With regards to the structural genes involved in anthocyanin biosynthesis, *CHS*, *F3′5′H*, *DFR*, *ANS* and *UFGT* were all more highly expressed in the purple parts of the peels (Figure 2C), suggesting upstream regulators (most probably TFs) may exist in the purple peels in response to UV-B exposure.

### 2.4. Interactions between TFs and Anthocyanin Biosynthesis Structural Genes

**Figure 3:**
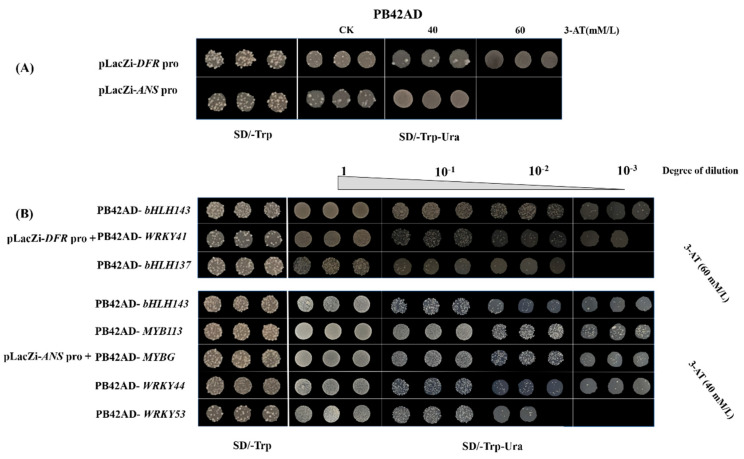
CabHLH143, CaWRKY41 and CabHLH137 interacted with the promoter of *CaDFR*, and CabHLH143, CaMYB113, CaMYBG, CaWRKY44 and CaWRKY53 interacted with the promoter of *CaANS*. (**A**) Growth conditions of PLacZi-*DFRpro* + PB42AD and PLacZi-*ANSpro* + PB42AD on SD/-Trp and growth conditions on SD/-Trp-Ura of different concentrations of 3-AT. (**B**) The interaction between *CaDFR* and anthocyanin-biosynthesis-related TFs and between *CaANS* and anthocyanin-biosynthesis-related TFs in yeast cells, respectively. Four black squares of SD-Trp-Ura (from left to right) represent four dilution concentrations. There are basically three replicates for each concentration of each combination.

In order to verify whether the selected TFs participated in the regulation of UV-B-induced anthocyanin biosynthesis, 50–2000 bp upstream of the transcription start site of anthocyanin structural genes and coding regions of TFs were analyzed. As shown in Supplementary Table S4, MYB113, WRKY53, MYB6, MYBG and MYB3 were recorded in the database and predicted to bind with promoters of anthocyanin biosynthesis structural genes. In order to verify the interactions that were predicted in silico, Y1H was performed between the TFs and anthocyanin biosynthesis structural genes. In the pre-experiment, 40 mM/L and 60 mM/L of 3-Amino-1,2,4-triazole (hereafter 3-AT) were confirmed to be sufficient to inhibit the self-activation of PLacZi-*DFRpro* and PLacZi-*ANSpro*, respectively (Figure 3A). From the Y1H results, it can be concluded that CabHLH143, CaWRKY41 and CabHLH137 were able to bind to the promoter of *CaDFR*; CabHLH143, CaMYB113, CaMYBG, CaWRKY44 and CaWRKY53 were able to bind to the promoter of *CaANS* (Figure 3B).

### 2.5. CaMYB113 Directly Bound to CabHLH143 and CaHY5

**Figure 4:**
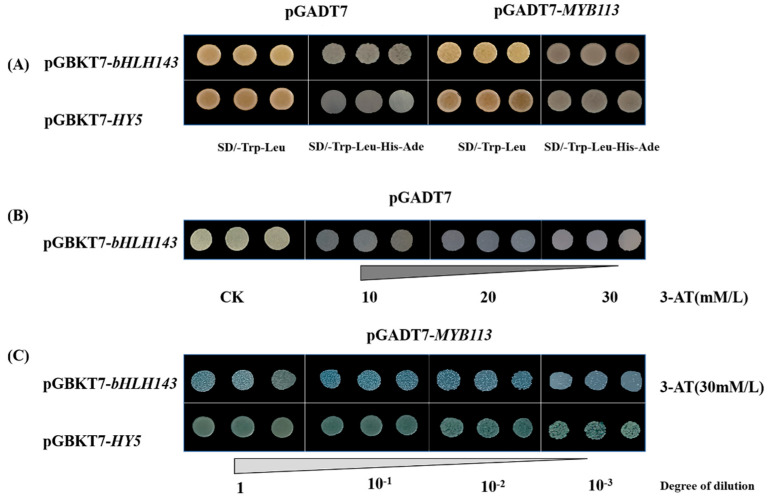
CaMYB113 interacted with CaHY5 and CabHLH143. (**A**) pGBKT7-*bHLH143* has self-activation on SD/-Trp-Leu-His-Ade, and pGBKT7-*HY5* does not. (**B**) Growth conditions of pGBKT7-*bHLH143* + pGADT7 on SD/-Trp-Leu-His-Ade with different concentrations of 3-AT. (**C**) CaMYB113 interacts with CabHLH143 and CaHY5 in yeast cells. The minimum concentration of 3-AT required to inhibit self-activation of pGBKT7-*bHLH143* is 30 mM/L. Four black squares represent four dilution concentrations. There are three replicates for each concentration of each combination.

To explore the interaction among the TFs, in silico predictions were performed via AraPPINet (https://netbio.sjtu.edu.cn/arappinet/) (accessed on 20 August 2021). As shown in Supplementary Table S5, bHLH137, MYBG, MYB113, WRKY53, MYB3 and MYB94 were predicted to have interactions with other TFs. There are some TFs, such as bHLH137, MYB113, WRKY53, MYB3 and MYB94, that may interact with HY5, a bZIP transcription factor, which plays a key role in the regulation of anthocyanin biosynthesis. However, HY5 was not significantly differentially expressed between the purple and the green peels (Figure 2C). In order to confirm the interactions mentioned above, Y2H was conducted. Results showed that CaMYB113 directly interacted with CaHY5 (Figure 4A,C). As to the interaction between CaMYB113 and CabHLH143, since CabHLH143 can be self-activated (Figure 4A), different concentrations of 3-AT were added to the media, and 30 mM was found to be the minimum effective dose (Figure 4B). Then, the interaction between CaMYB113 and CabHLH143 was confirmed (Figure 4C).

### 2.6. Function Analysis of CaMYB113 by VIGS

**Figure 5:**
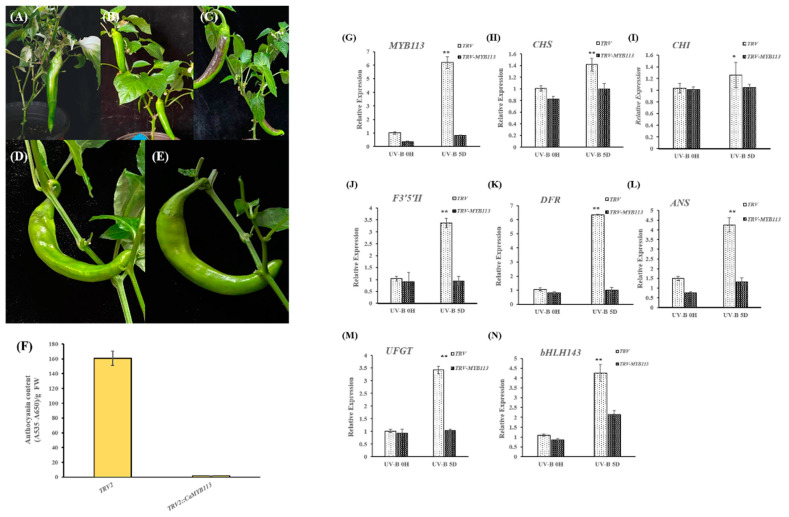
Analysis of VIGS of the *CaMYB113* gene. (**A**) White vertical stripes appear on the pepper fruit peel inoculated with TRV2-*PDS*. (**B**,**C**) Phenotype of pepper fruits inoculated with TRV2 as controls before treatment and UV-B-induced for 5 days. (**D**,**E**) Phenotype of pepper fruits inoculated with TRV2-*CaMYB113* before treatment and UV-B-induced for 5 days. (**F**) Anthocyanin contents in fruit peels between TRV2-inoculated and TRV2-*CaMYB113*-inoculated plants UV-B-induced for 5 days. Numerical values are presented as means (± SE) from three independent biological replicates. (**G**–**N**) Relative expression of *CaMYB113*, *CabHLH143* and anthocyanin structural genes in fruit peels of TRV2-inoculated and TRV2-*CaMYB113*-inoculated plants before treatment and UV-B-induced for 5 days, respectively. Statistically significant differences between purple zone and green zone determined by Student’s *t*-test. (** *p* < 0.01, * *p* < 0.05).

Since *CaMYB113* bound to the promoter regions of two anthocyanin structural genes and, at the same time, directly interacted with two TFs, further function analysis was performed by VIGS. Photobleaching was observed in the fruits of the positive control (TRV2::PDS + TRV1) 80 days after the injection, indicating that the VIGS system worked well (Figure 5A). Then, *CaMYB113*-VIGS and the negative control were treated with UV-B light for 5 days. After that, purple color was only discovered on the light-facing side of the negative control (TRV2 + TRV1) but not on the *CaMYB113*-VIGS fruits (Figure 5B–E). Consistent with the above observations, anthocyanin was significantly accumulated in the light-facing side of the negative control fruits but not in the same part of the *CaMYB113*-VIGS fruits (Figure 5F). qRT-PCR results showed that the expression of *CaMYB113* was significantly induced by UV-B treatment on the light-facing side of negative control fruits but stayed at low levels in that of the *CaMYB113*-VIGS fruits (Figure 5G). Later, more qRT-PCR analyses were performed to test the expression changes of the genes involved in anthocyanin biosynthesis. As shown in Figure 5H–N, although a significant increase in *bHLH143* expression was observed on the light-facing side of the *CaMYB113*-VIGS fruits, silencing of *CaMYB113* still significantly inhibited the upregulation of the expression of *CHS*, *CHI*, *F3’5’H*, *DFR*, *ANS*, *UFGT* and *bHLH143*, which was induced by UV-B, suggesting that CaMYB113 may be a key regulator involved in controlling UV-B-induced anthocyanin biosynthesis.

## 3. Discussion

### 3.1. UV-B Effectively Induced Anthocyanin Biosynthesis in the Peel of 19Q6100 Fruit

Fruit color is an important trait for horticultural produce, including peppers. As to the pepper varieties collected and sold at the mature green stage, the green color of the peel determines the final price of the commodity. However, the sun-facing side of the fruit peel of some varieties accumulates anthocyanin. This trait severely impacts the value of the commodity, as well as the income of the pepper growers. Although the abovementioned trait is important for the pepper industry, very few studies have focused on this issue. In contrast, many researchers have made efforts to disclose the mechanism of consistently purple fruits at the mature green stage [38,40,41,42]. In this study, a natural mutant was discovered in our experimental field, which bears fruits that partially turn purple when exposed to sunlight. Light is an important environmental factor that regulates anthocyanin biosynthesis. Moreover, ultraviolet light has been proven to have more profound effects and significantly increase anthocyanin contents in apple, tomato, peach and strawberry fruit [23,24,25,26,43]. Therefore, through treatments with different wavelengths of light, UV-B was confirmed to effectively induce the partial purple peel phenomenon in the fruit of 19Q6100, an inbred line derived from the mutant mentioned above. The UV-B-induced anthocyanin biosynthesis pathway was clarified, starting with the reception of UV-B by the UVR8 photoreceptor. After that, UVR8 interacts with COP1 and reduces the antagonistic effect of COP1 on HY5, which leads to the upregulation of the expression of *HY5* [44]. HY5, on one hand, may directly bind to the promoters of anthocyanin structural genes and promote the accumulation of anthocyanins; on the other hand, it may bind to the promoters of MYBs, which could later regulate the expression of other TFs, as well as the expression of anthocyanin structural genes [26,36,45]. In this study, UVR8 was discovered to be significantly differentially expressed between the light-facing and the shaded side of the fruit, according to transcriptome analysis. Although a similar phenomenon was not observed for the *HY5* gene, we found that it had the same expression trend as UVR8, according to qPCR (Supplementary Figure S1). We speculated that since expression of HY5 was only analyzed at one point in time, the change in expression can simply be ignored.

### 3.2. Upstream Regulators May Be Involved in UV-B-Induced Anthocyanin Accumulation in 19Q6100 Fruit

Transcriptome analysis revealed that the activity of many anthocyanin biosynthesis structural genes was significantly increased in the purple peel, based on which it can be presumed that upstream regulators may be involved in this regulation. Therefore, some anthocyanin-related transcription factor genes were identified among the DEGs, including *CaMYB113*, *CabHLH137*, *CabHLH143*, *CaMYB6*, *CaMYB3*, *CaMYB94*, *CaMYBG*, *CaWRKY41*, *CaWRKY44* and *CaWRKY53*. Previously studies reported that *MYB6* was involved in the promotion of anthocyanin and pro-anthocyanidin biosynthesis in Populus tomentosa [46], and *MYB3* was a transcriptional repressor of anthocyanin biosynthesis in citrus fruit [47]. *WRKY44* was able to strongly activate the promoters of the *F3’H* and *F3’5’H* genes in kiwifruit [48]. *WRKY41* in *Brassica napus* had a similar role with AtWRKY41 in regulating anthocyanin biosynthesis when overexpressed in *A. thaliana* [49]. Consistent with these results, in this study, Y1H confirmed that seven of the abovementioned TFs bound to the promoters of anthocyanin biosynthesis structural genes: CaMYB113, CabHLH137, CabHLH143, CaMYBG, CaWRKY41, CaWRKY44 and CaWRKY53. This suggests that these seven TFs may be upstream regulators of anthocyanin biosynthesis.

### 3.3. The HY5-CaMYB113-CabHLH143 Pathway

Among the seven TFs, CaMYB113 interacted with both anthocyanin biosynthesis structural genes and other transcription factors. CaMYB113 is a homolog of AtMYB113 that has been reported to be involved in the regulation of anthocyanin biosynthesis in many plant species [11,13,50,51]. Meanwhile, this TF is also a member of the MBW complex. In pepper, *CaMYB113* was reported to underly a locus on chr10 that controls the consistent purple trait of the mature green fruit [40]. In this study, CaMYB113 was found not only to bind to the promoters of the downstream anthocyanin structural genes, *ANS*, but also interacted with CaHY5 and CabHLH143 at the protein level, suggesting CaMYB113 may be a key regulator of UV-B-induced anthocyanin accumulation. Moreover, silencing of *CaMYB113* led to the inhibition of UV-B-induced anthocyanin accumulation, as well as the related gene expression, which further confirmed the importance of this TF in the process. CabHLH143 is a member of the bHLH transcription factor family. However, interactions and functions of this TF have not been well studied in Arabidopsis and other model plants. In this study, CabHLH143 was differentially expressed between the purple and green peels. Meanwhile, Y1H and Y2H indicated that this TF interacted with CaMYB113, as well as two anthocyanin biosynthesis genes, *CaDFR* and *CaANS*, indicating an important role of CabHLH143 in the regulation of UV-B-induced anthocyanin accumulation.

In the anthocyanin studies, the MBW transcriptional complex was considered to be a critical regulator that determines anthocyanin biosynthesis. Many kinds of environmental and developmental signals, such as light, temperature and endogenous hormones, regulate the formation and activation of the MBW complex. In this study, a protein–protein interaction was detected between CaMYB113 and CabHLH143, which seemed to form an MYB complex with an unknown WD TF and, ultimately, a response to UV-B via interaction with HY5.

**Figure 6:**
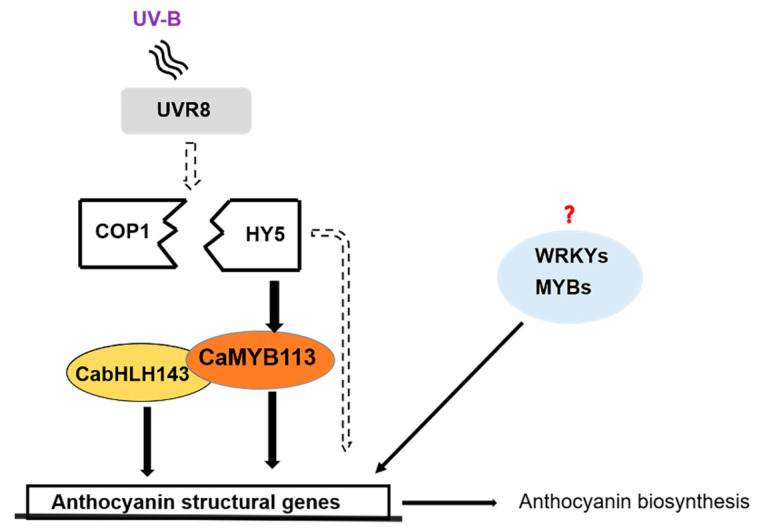
A hypothetical model of UV-B-induced anthocyanin biosynthesis in pepper fruit. A UV-B signal activates the UVR8 receptor-forming monomer, which binds to the COP1 protein and releases HY5. HY5 not only binds directly to the anthocyanin structural genes but also upregulates the expression of CaMYB113. CaMYB113 and CabHLH143 may regulate the expression of several anthocyanin structural genes by forming a complex. The above processes ultimately promote the accumulation of anthocyanins. In addition, some TFs that may be involved in the regulation of anthocyanin structural genes still need to be studied in depth. (The solid line represents the results of this study, and the dotted line represents those of previously reported studies.).

An HY5-CaMYB113-CabHLH143 pathway may exist in the UV-B-induced anthocyanin accumulation process in the fruit peel of 19Q6100 that not only connects the upstream UVR8-COP1-HY5 UV-B signal-transduction pathway but also links the downstream TFs and anthocyanin structural genes, ultimately regulating UV-B-induced anthocyanin biosynthesis and accumulation (Figure 6).

## 4. Materials and Methods

### 4.1. Plant Materials and Growth Conditions

The C. annuum inbred line “19Q6100” was used as the material in this study, provided by College of Horticulture, China Agricultural University. From the seedling stage to the fruiting stage, the pepper plants were put in a growth chamber under conditions of 27 °C, under red-blue light for 16 h and 16 °C, darkness for 8 h, respectively. During the mature green period of fruits, the control group continued to grow under red-blue light, and one side of the fruit could be illuminated by the light source without being blocked. For the treatment group, UV-B light (40 μW/cm^2^) was added on the base of the control group. Samples of fruit treated for at least 5 days with mixed purple and green peels were selected for anthocyanin determination, RNA extraction and qPCR analysis. Three biological replicates were performed for each sample.

### 4.2. Anthocyanin Extraction and Determination

Anthocyanin extraction and determination was conducted as previously described [52]. In short, 1 g fresh weight (FW) of fruit peel was transferred into a tube filled with 4.3 mL of extraction solution (1-propanol/HCl/distilled water, 18/1/81, *v*/*v*/*v*). The tubes were placed in boiling water for 6 min, then incubated overnight at room temperature in the dark. Another 3.7 mL of extraction solution was added to the mixture, then centrifuged at 1000× *g* for 5 min. After the supernatant was filtered by a 0.45 μm filter (Millipore, Burlington, MA, USA), the OD values of A535 and A650 were read by spectrophotometer, and the anthocyanin content in the extract was quantified (A535-A650) per gram FW. Three biological replicates were performed for each analysis.

### 4.3. RNA Extraction, cDNA Synthesis and Real-Time PCR Analysis

Samples were collected from the purple and green peels of the same fruits and immediately frozen in liquid nitrogen. Three biological replicates were performed for each sample, and each replicate peel was collected from 3 different plants. Total RNA was extracted from samples using the quick RNA isolation kit (Huayueyang, Beijing, China) and following the manufacturer’s protocols. cDNA was synthesized from 1 μg RNA using HiScript^®^ III 1st Strand cDNA Synthesis Kit (Vazyme, Nanjing, China). Primers used for the qRT-PCR were designed online on the NCBI website (https://www.ncbi.nlm.nih.gov/tools/primer-blast/) (accessed on 15 October 2021) and are shown in Supplementary Table S6. qRT-PCR was performed with a ChamQ Universal SYBR qPCR Master Mix (Vazyme, Nanjing, China) on an ABI 7500 real-time PCR system (Applied Biosystems, Foster City, CA, USA) under the following conditions: 95 °C for 30 s, 40 cycles at 95 °C for 5 s and 60 °C for 30 s. A pepper gene, *UBI* (*Capana06g002873*), was used as the reference. The 2^−ΔΔCt^ method was used to calculated relative expression of each gene [53]. Three biological replicates and three technical replicates were performed for each sample. 

### 4.4. RNA-Seq Analysis

Purple and green peels from the same fruits were collected as samples for RNA-seq. Fruit peels were carefully cut off with a scalpel, then quickly frozen in liquid nitrogen and stored at −80 °C. Transcriptome libraries were constructed using an NEBNext^®^ UltraTM RNA Library Prep Kit for Illumina^®^ (NEB, San Diego, CA, USA) following the manufacturer’s protocols. Three biological replicates were performed for RNA transcriptome analyses. Then, the library was sequenced on an Illumina Hiseq 4000 platform, and 150 bp paired-end reads were generated.

Adapter, ploy-N, and low-quality reads were removed from raw data by QC before downstream analyses. After read filtering, the Q20, Q30 and GC contents of the clean data were calculated to ensure downstream analyses were based on high-quality clean data. Clean reads were mapped to reference gene and reference genome by TopHat2 [54]. The transcript levels for genes were calculated and transformed to fragments per kilobase of transcript per million mapped reads (FPKMs) by Cuffquant and Cuffnorm (v2.2.1). DEGs were identified based on a comparison of FPKMs between different samples under the thresholds of false discovery rate ≤ 0.001, *p*-value ≤ 0.01 and absolute Log2 fold change value ≥ 1 using the DESeq2 R package [55]. Gene Ontology (GO) classification and functional enrichment were analyzed using TBtools [56].

The RNA-Seq raw sequence data are deposited in the Short Read Archive (SRA) of NCBI and are available with accession number PRJNA790487.

### 4.5. Yeast One-Hybrid Assay

In order to investigate whether the transcription factors bound to the promoters of anthocyanin biosynthesis structural genes which were analysis was conducted by JASPAR (https://jaspar.genereg.net/) (accessed on 20 August 2021) [57]. 50–2000 bp upstream of the transcriptional start site of anthocyanin structural genes were amplified and inserted into the vector PLacZi as bait sequences. The coding regions of the transcription factors were amplified and inserted into the vector PB42AD as prey protein. Primers used for amplified and plasmid construction are listed in Supplementary Table S6. The specified combinations of constructs were co-transformed into yeast strain EGY48. The minimal inhibitory concentration of 3-Amino-1,2,4-triazole(3-AT) was detected by the empty pB42AD vector plus PLacZi promoters. After 3 days of transformation, 3 yeast clones grown on the SD/-Trp-Ura (SD-TU) medium supplemented of each combination were picked and diluted proportionally to different concentrations, and the culture was inoculated on SD-TU medium at 30 °C for 3 days.

### 4.6. Yeast Two-Hybrid Assay

Y2H studies were performed as reported, with some modifications [58]. Predictions of the interactions between TFs were performed by AraPPINet (https://netbio.sjtu.edu.cn/arappinet/) (accessed on 20 August 2021) [59]. The coding regions of the targets, including MYB113, bHLH143, HY5, were amplified and inserted into pGADT7 and pGBKT7 vectors, respectively. Primers used for amplified and plasmid construction are listed in Supplementary Table S6. The constructs were co-transformed into yeast strain Y2HGold with the different combinations and then cultured on SD/-Leu-Trp (SD-LT) medium supplemented at 28 °C for 2–3 days. The minimal inhibitory concentration of 3-AT was detected by the empty pGADT7 vector plus pGBKT7-TFs, then transferred to combinations and diluted proportionally to different concentrations onto SD/-Leu-Trp-His-Ade with 4 mg/mL X-α-gal to determine the interactions between bait and prey proteins. 

### 4.7. VIGS

Taking ‘Zunla 2.0′ genome as reference, the specific coding regions of the *CaMYB113* gene were confirmed for VIGS vector construction by the basic local alignment search tool (BLAST) on SGN (https://vigs.solgenomics.net/) (accessed on 11 September 2021) and used for silencing analysis [60]. A ~150 bp fragment of the coding region of *CaMYB113* was inserted into the TRV2 vector by double-enzyme digestion to construct the TRV2::*CaMYB113* vector. Additionally, TRV2::*CaPDS*, as a positive control vector, was constructed by inserting a fragment of ~100 bp of the coding region of *CaPDS* gene into TRV2. The two enzyme-restriction sites were XbaI and BamHI. Primers used for amplified and plasmid construction are listed in Supplementary Table S6. The constructed TRV2 vectors and TRV1 vector were transformed into *A. tumefaciens* (strain GV3101). Resuspending TRV1 with TRV2::*CaMYB113*, TRV2 (as a negative control) and TRV2::*CaPDS* in liquid induction medium and mixing at 1:1 ratio, OD600 = 1.0, respectively. After resuspension liquid was placed in darkness for 8 h, seedlings of 19Q6100 pepper plants at the two-leaf stage were injected. The injected seedlings were grown in a growth chamber at 22 ± 2 °C and 16 h of light/8 h of darkness, and relative humidity was controlled in the range of 80 ± 5%.

## 5. Conclusions

In this study, we used 19Q6100 as material, of which some part of the peel could accumulate anthocyanin by in response to UV-B exposure. A total of 108 DEGs were identified at transcriptomic levels. Y1H results suggested that CabHLH143, CaMYB113, CaMYBG, CaWRKY44, CaWRKY53, CaWRKY41 and CabHLH137 were able bind to the promoter of anthocyanin structural genes. The interactions among CaMYB113, CabHLH143 and CaHY5 were verified by Y2H. The function of *CaMYB113* in UV-B-induced anthocyanin biosynthesis in pepper was discovered. Based on our results, we propose a hypothetical model of UV-B-induced anthocyanin biosynthesis in pepper fruit (Figure 6). This study could deepen our understanding of the regulatory mechanism of anthocyanin biosynthesis in pepper and also provide useful information for the future study of UV-B-induced anthocyanin accumulation in fruit peel of other species.

## Data Availability

RNA-Seq raw sequence data were deposited in the Short Read Archive (SRA) of NCBI and are available with accession number PRJNA790487 (https://www.ncbi.nlm.nih.gov/bioproject/PRJNA790487) (accessed on 18 December 2021).

## Supplementary Materials

The following supporting information can be downloaded at: https://www.mdpi.com/article/10.3390/ijms23041960/s1.
