# Supplementary material for: Identification of the Regulatory Genes of UV-B-Induced Anthocyanin Biosynthesis in Pepper Fruit
Source: Int J Mol Sci. 2022 Feb 10;23(4):1960. doi: 10.3390/ijms23041960 (PMC8879456; doi:10.3390/ijms23041960)
Supplement: Supplementary file 1 [file ijms-23-01960-s001.zip › ijms-1557696-supplementary.pdf]

Table S1

Sequence comparison result between sequencing data and reference genome

IDs	Total Reads	Mapped Reads
Green1	41,042,328	35,461,163 (86.40%)
Green2	41,856,712	38,610,947 (92.25%)
Green3	41,317,242	38,729,423 (93.74%)
Purple1	44,010,120	41,023,566 (93.21%)
Purple2	42,963,496	39,564,088 (92.09%)
Purple3	40,870,712	37,751,155 (92.37%)

Table S2

The correlation coefficients (R2) between the biological replicates

	Green1	Green2	Green3	Purple1	Purple2	Purple3
**Green1**	1	0.935	0.932	0.912	0.920	0.887
**Green2**	0.935	1	0.883	0.925	0.919	0.901
**Green3**	0.932	0.883	1	0.904	0.858	0.916
**Purple1**	0.912	0.925	0.904	1	0.922	0.933
**Purple2**	0.920	0.919	0.858	0.922	1	0.954
**Purple3**	0.887	0.901	0.916	0.933	0.954	1

Table S3

Significantly differential expressed genes

#ID	Green01	Green02	Green03	Purple01	Purple02	Purple03
Capana00g000451	13.853798422924	10.490900850018	14.188315839846	1.385612959518	0.779435944301	1.038474627512
Capana00g000469	8.668486040113	6.581850449947	4.221050090869	1.592169395711	1.308666847792	1.288908830393
Capana00g002009	0.757007074139	0.831810163552	1.572141480127	7.239404095021	9.148326952471	7.022934394304
Capana00g002054	5.908919010396	8.112961673871	15.175854271058	94.689584799659	94.574907919453	67.928083615868
Capana00g002089	80.712789597493	44.914247627937	37.854774938462	6.328510478278	2.179610395912	1.709111927491
Capana00g002532	1.177128042634	1.229775023937	1.115135607645	1.896195518042	2.110716857157	2.421150623011
Capana00g003106	58.784102673778	57.877724082257	86.093913774407	17.096013429652	20.666737067436	30.940832272725
Capana00g003196	8.674988859286	7.075938923868	7.959511038726	2.734875105528	2.436452457119	3.091245186819
Capana00g003394	0.049432954792	0.643803395373	0.900013935597	12.521469071632	6.392436777471	10.955975991677
Capana00g003926	1.044814540055	1.444619618387	17.345190744458	0.080972633897	0.097849276017	1.617645988834
Capana00g003936	9.874596757075	1.288242576081	3.923280710488	0.522972614417	0.189726534607	0.669724405584
Capana00g004656	9.650505493144	11.790581942890	9.371696570516	5.685196655173	3.111486238307	2.352901265452
Capana01g001736	31.069056412698	65.111530099267	18.121714770453	0.893729144003	0.756909647680	1.083226924885
Capana01g002564	0.331414698071	0.303888043572	0.333705374883	6.487167343159	12.209463794271	17.531143457828
Capana01g002820	67.616327187545	56.655246867745	118.391804682659	32.283148268109	20.382520232688	29.151753488128
Capana01g002824	13.818114690068	13.168155046166	25.259362733786	5.891509394644	5.696976938318	7.869175534793
Capana01g003071	14.826057512031	5.076876616364	13.860698256865	2.362187075912	0.505403259178	1.931888014938
capana01g004167	25.628118366394	26.176609376962	27.630448237023	2.678430521731	2.707285164252	3.167228575458
Capana01g004472	2.306245498181	1.761446417743	2.122503830993	6.498418399345	9.123387078715	8.575408976301
Capana02g000203	2.565192559182	4.217034594488	1.842144685598	7.736588257588	8.420933366369	7.592393113114
capana02g000906	1.548537080077	1.545063600877	1.747363650143	8.229100137664	7.248717389649	7.758974371786
Capana02g002029	5.025078340635	2.958708943843	4.414919911339	0.070348049484	0.077232752693	0.092262790518
Capana02g002150	0.173542426626	0.030489639395	0.266423559898	6.696379827733	5.667888593024	2.924856751989
Capana02g002314	7.490679407367	8.016390499963	14.813995345229	1.522055458463	0.760249582011	1.831153555598
Capana02g002342	1.135526795064	2.241647007294	1.163168401314	13.461286525801	21.636547003253	9.769631637967
Capana02g002763	1.965576797586	2.626415919077	2.981950315862	184.686316672219	125.959814863192	131.494736617403
Capana02g003258	0.738074593457	1.281353480595	0.680021854077	0.174958661964	0.149518751357	0.127267572721
Capana03g000766	6.984039397275	5.316090067397	3.426914125929	17.065610600251	17.865072320939	20.588876410864
Capana03g001190	0.333511253646	0.095576328504	0.617740269926	4.713283024935	6.401819732647	26.461337722476
Capana03g001474	6.942207205211	14.494453866603	7.649219795255	2.194809702043	2.421265344157	1.742439891231
Capana03g001546	7.033750829129	6.470304766125	6.567445125037	75.910039382388	75.888540213845	42.720619763001
Capana03g001564	1.027525416804	0.903230108452	1.036469207234	0.398955430278	0.822046780027	3.813779302363
Capana03g001619	3.526286642774	3.267564183113	2.277721135198	13.515286833451	19.597332524346	14.120778750703
Capana03g003255	0.549840768688	1.188784623121	0.435526536694	7.699243817356	8.678007116046	8.540732268894
Capana03g003445	0.225601527121	0.394221712215	1.920306915508	11.308047402550	11.164794129775	8.677135078501
Capana03g004041	6.499788962482	8.071744448121	3.520993990607	23.769279528905	24.906639819168	46.575931548871
Capana03g004110	1.818231256126	2.137509875414	9.604538997421	0.221852384883	0.105814964284	0.587129667203
Capana03g004255	0.482610876279	0.880687308687	1.190029039649	6.021746924431	17.305686836187	119.943548217941
Capana03g004445	2.950102254053	1.356801085556	3.702865264295	7.837755384109	8.870957257854	13.918080378377
Capana03g004562	1.086247036138	0.362456340589	2.093800136871	22.381247477217	25.420575738416	122.097700338243
Capana04g000944	2.192197691858	3.616661641213	1.515613006671	5.156711643953	8.332606066034	11.332642053552

Capana04g001651	0.088174555692	0.301414019158	0.097210246321	7.747434762325	57.559040098346	16.611916447289
Capana04g001985	0.489537558308	0.741359466205	0.841747070813	2.236384282032	1.389446873282	1.439597792041
Capana04g002426	0.656306880378	0.358627536533	0.544446610772	2.622083989163	2.174303339294	2.026436329939
Capana05g000399	0.996523426301	1.846955612726	0.713314499771	4.676336041167	2.688514737576	5.039043228189
Capana05g001205	0.515033820794	0.057560354749	0.874596377567	5.508657507174	5.144603844413	7.200091454493
Capana05g002190	3.208407102841	3.559561461457	2.458311826436	11.954677422623	15.913128105334	13.910987793709
Capana05g002274	2.106045526956	3.346786665472	2.822167276143	4.630941839019	4.547820046711	6.216322021975
Capana06g000044	2.156859383757	0.660468092124	0.671391842867	5.289520355180	19.941167910674	23.554575903458
Capana06g000103	1.152957737918	2.392432240637	1.241834846025	7.759387542921	8.718205905295	4.183943520412
Capana06g000202	6.238094783224	4.549354626277	2.579355900184	11.338456292213	10.442179682972	9.058686360937
Capana06g001010	1.442790263507	6.547924541368	0.722805697685	12.252383931674	8.463125265723	36.023772511717
Capana06g002713	1.548326266335	2.509535263691	2.767349814778	5.071487429970	6.769813662523	9.717303049022
Capana06g002887	1.215803581085	2.604152359612	1.457940512877	14.634270509597	15.475724473962	11.782073648426
Capana07g000347	1.574674405575	2.226947261884	1.369758429642	9.506716706274	12.072380709634	6.054579054355
Capana07g000730	1.399693250684	2.641215179213	1.257365452188	3.955429139434	7.427005109926	6.917033741494
Capana08g000946	1.023496004034	0.336768830947	0.260879144757	17.701213722421	25.050876343018	12.463951688668
Capana08g000947	1.403571965594	2.476582920799	1.658126934871	3.823599077794	6.131274807006	7.228389003469
capana08g001044	4.692055412333	5.714532608872	6.750186726052	18.905256267059	13.061551309819	14.949868473011
Capana08g001296	4.034051584378	3.259015557998	1.502655999142	15.090419114777	6.774123356862	5.471858207572
Capana08g001504	0.053857644259	0.206259245408	0.057684979738	1.050548689048	0.972246667703	0.781435192436
Capana08g001584	2.253584448212	1.746853560684	6.053747826861	15.845951353499	13.925216978352	12.123858145583
Capana08g002528	17.876638875725	17.929179860213	14.744215851434	6.388185041856	8.224943019616	10.587520611835
Capana08g002542	0.857576254531	0.545023383627	0.329975783944	1.487038349908	1.911237771635	1.520121382775
Capana09g000136	0.871545340847	0.154533471968	0.139530863777	18.590061206423	18.867246071928	12.537411488541
Capana09g000318	4.863003833359	5.213516570529	3.833505891039	13.909086920291	12.349279755442	29.054518460836
Capana09g001532	0.250816365056	0.742018566117	0.654184322348	10.298505116925	31.997161033673	1.052724480599
Capana09g001714	38.200767619684	56.368623605897	78.708407499061	3.292803095425	6.348501057726	4.044075953935
Capana09g001762	18.387568856439	22.770659083666	98.883190506638	1.188481214175	0.487954075999	1.350650415609
Capana10g000618	3.515701114238	4.769394838083	2.269749243649	15.554941412825	12.535816401359	4.050583241674
Capana10g001433	1.302843340758	1.532779089596	1.350592341526	44.025496328755	25.744239031501	36.221239177909
Capana10g001637	7.403032127312	8.273924618103	5.049475233098	0.083027593078	0.172217272039	0.168202194475
Capana10g001654	1.500693214925	3.731499255878	2.659316299849	32.954502757604	35.865208209031	13.272508337573
Capana10g001655	7.004049003841	4.936448121409	4.049113120713	0.206158915757	0.243399582871	2.138379898469
Capana10g001683	4.213383538103	8.448530703069	4.720296784999	17.888418409684	24.427363936854	22.760775199918
Capana10g001791	0.735836920813	0.674530471159	0.660393335444	2.647377601767	1.988199133913	1.841623572903
Capana10g001978	2.244778504173	1.497408808914	2.012934843129	19.978970831024	16.356582441039	28.522459266114
Capana10g002161	5.973630864995	5.857487085064	8.007582747966	1.837282887962	2.351785905061	3.240046245158
Capana11g000195	2.446106907915	2.053525634066	2.241911093933	15.832645146392	11.185039308174	8.087049892495
Capana11g000434	20.025088965554	59.824894174639	11.226842664916	0.754864939522	0.545517510688	0.159827298457
Capana11g001860	15.032083249409	10.217738870711	21.137493833572	1.182089082443	1.885977395653	2.973196098754
Capana12g000116	35.966075936714	60.910834479576	65.145449312907	7.634184970227	11.452124488191	17.198655950836
Capana12g000713	3.386012732105	2.616439457924	3.764979475711	6.920217229751	9.706091628002	10.522485124122
Capana12g000836	8.859702615049	3.706822969095	5.739518812684	18.189809196053	29.867355862158	42.365324498189
Capana12g000885	1.294002859698	1.389410863259	1.407962855993	20.211446034328	15.213283964813	21.479494928869

Capana12g001364	1.976898433052	1.059751081585	1.616738939055	0.161975781774	0.129621928542	0.581978507814
Capana12g001653	0.145930858899	0.665182151576	0.574354029179	2.282101829121	5.718457310645	3.816816618549
Capana12g002517	2.083010921136	4.554703864907	2.386183133489	12.693411393346	26.642381311731	8.425897544784
Capsicum_annuum_newGene_1026	5.122920706853	9.474358616548	11.411968194637	24.990635416587	23.974432663187	56.260961876803
Capsicum_annuum_newGene_11978	1.528216479295	3.743822403151	1.540566099286	13.622722132629	13.899272669952	11.776404596635
Capsicum_annuum_newGene_13276	0.119962168557	0.174225913189	0.134321814226	1.221847810354	2.587403439631	2.367224705599
Capsicum_annuum_newGene_13449	1.370547004676	4.768954960478	1.940476580165	28.537995080179	14.154769456197	26.593183331711
Capsicum_annuum_newGene_13937	11.380262505341	19.887095729082	7.268582348498	86.489622618058	42.173675089387	95.595896635455
Capsicum_annuum_newGene_14030	1.772927698612	0.345432487737	1.525869318499	14.874838391819	12.396832220092	7.944928807851
Capsicum_annuum_newGene_147	1.971208813788	1.823236525196	2.450367423255	39.822425534288	25.059364558584	38.044298851531
Capsicum_annuum_newGene_1883	2.953690665921	3.826207684907	3.101864969225	10.858009890107	10.048357479185	12.704521010457
Capsicum_annuum_newGene_19755	2.959477329438	3.630153426624	0.493233727386	23.145961364012	43.745788613329	9.019995809044
Capsicum_annuum_newGene_19818	4.497668814641	1.704890140544	2.753873410774	0.020332948493	0.148817793768	0.116512961983
Capsicum_annuum_newGene_21271	16.853314092361	19.891005109399	16.893702123086	1.830211757349	2.235233856193	2.897799820677
Capsicum_annuum_newGene_21328	6.338473527784	2.713089267818	3.647001143962	0.164806248597	0.370127355311	0.687093580585
Capsicum_annuum_newGene_220	3.292386539426	1.613222560509	25.395132444229	0.439821533423	0.285402336586	0.425660039347
Capsicum_annuum_newGene_3310	23.527189092246	27.443162949629	4.664544696154	1.512702728996	0.901252308934	0.890686282675
Capsicum_annuum_newGene_5451	0.362182497114	0.911453362071	0.804000827048	2.976392034651	5.470916226575	3.782914494195
Capsicum_annuum_newGene_5510	4.254123092405	4.319313255204	17.557879931268	2.380273545526	0.176629408246	1.032520636101
Capsicum_annuum_newGene_6909	9.180454034598	10.127595674892	16.413327406353	1.163294380293	0.868848991777	2.200350684896
Capsicum_annuum_newGene_7253	1.123842605636	1.740995576687	1.130437901326	7.005269653388	14.937241094345	22.560365250948
Capsicum_annuum_newGene_9008	3.180542007722	3.776454097026	4.216058319185	8.069858228016	9.606964941945	10.592019324945
Capsicum_annuum_newGene_9477	0.681092524246	0.221403597849	0.525572867704	1.130777571016	3.596258692903	2.426272744329

Table S4

Prediction of interaction between TFs and anthocyanin biosynthesis genes

Matrix ID	Name	Score	Relative score	Sequence ID	Start	End	Predicted sequence
MA1181.1	MA1181.1.MYB113	11.945911	0.938937922	*CHS*	532	542	TTTTTAGTTAT
MA1181.1	MA1181.1.MYB113	11.195956	0.925675195	*CHS*	1828	1838	CAGTTTGTTAT
MA1181.1	MA1181.1.MYB113	13.167757	0.960545887	*DFR*	1160	1170	AAGTTCGTTAG
MA1181.1	MA1181.1.MYB113	8.673596	0.881068037	*DFR*	747	757	ATATTAGTTGT
MA1181.1	MA1181.1.MYB113	12.058535	0.940929629	*ANS*	1801	1811	TAATCCGTTAC
MA1181.1	MA1181.1.MYB113	8.520201	0.878355282	*ANS*	144	154	GTTGTAGTTAT
MA1181.1	MA1181.1.MYB113	8.324994	0.874903112	*ANS*	296	306	AAGTTCCTTAT
MA1181.1	MA1181.1.MYB113	7.9939847	0.869049313	*ANS*	510	520	AATTCAGATAT
MA1181.1	MA1181.1.MYB113	6.8210897	0.848307026	*ANS*	922	932	AAGATTGTTGC
MA1181.1	MA1181.1.MYB113	6.648202	0.845249558	*ANS*	496	506	TTTGTGGTTAT
MA1181.1	MA1181.1.MYB113	6.1335855	0.836148724	*ANS*	377	387	TTGTTAGTCAT
MA1181.1	MA1181.1.MYB113	6.082975	0.835253692	*ANS*	558	568	TATTTAATTAC
MA1181.1	MA1181.1.MYB113	11.645147	0.933619002	*UFGT*	949	959	TTATTAGTTAT
MA1181.1	MA1181.1.MYB113	8.924697	0.885508673	*UFGT*	869	879	ATTATTGTTAT
MA1181.1	MA1181.1.MYB113	8.293036	0.874337951	*UFGT*	1188	1198	TAAGCCGTTAG
MA1181.1	MA1181.1.MYB113	7.6230617	0.862489653	*UFGT*	1142	1152	AAATTAATTAT

MA1181.1	MA1181.1.MYB113	6.8776107	0.849306582	*UFGT*	234	244	ATGTCAATTAT
MA1181.1	MA1181.1.MYB113	6.5937414	0.844286441	*UFGT*	67	77	CGATCCGTTAG
MA1181.1	MA1181.1.MYB113	6.5739284	0.843936053	*UFGT*	1454	1464	GATTTCATTAT
MA1181.1	MA1181.1.MYB113	6.0043473	0.833863187	*UFGT*	125	135	TAGATAGATAT
MA1181.1	MA1181.1.MYB113	5.8783383	0.831634757	*UFGT*	371	381	AATATAATTAT
MA1805.1	MA1805.1.WRKY53	8.278576	0.88672152	*CHS*	582	590	GGTCAATAA
MA1776.1	MA1776.1.WRKY53	8.169707	0.884047112	*DFR*	849	857	AGTCAAAAC
MA1776.1	MA1776.1.WRKY53	7.050233	0.856546695	*DFR*	893	901	GGTCAAGAT
MA1776.1	MA1776.1.WRKY53	5.223036	0.811660739	*DFR*	56	64	AGTAAATTC
MA1776.1	MA1776.1.WRKY53	5.177666	0.810546212	*DFR*	755	763	TGTCAAAAG
MA1776.1	MA1776.1.WRKY53	5.103447	0.808722982	*DFR*	1987	1995	GGTAAAAGA
MA1776.1	MA1776.1.WRKY53	5.1020756	0.808689293	*DFR*	274	282	AGTCTATTC
MA1776.1	MA1776.1.WRKY53	8.284378	0.886864053	*ANS*	1186	1194	TGTCAACTT
MA1776.1	MA1776.1.WRKY53	6.7486296	0.849137668	*ANS*	544	552	AGTCTACTT
MA1776.1	MA1776.1.WRKY53	6.1264787	0.833854238	*ANS*	1055	1063	GGTCTATGG
MA1776.1	MA1776.1.WRKY53	5.5839386	0.820526486	*ANS*	787	795	GGTCTAAGC
MA1776.1	MA1776.1.WRKY53	5.3912582	0.815793202	*ANS*	1570	1578	ATTCAACCA
MA1776.1	MA1776.1.WRKY53	5.212273	0.811396348	*ANS*	652	660	CTTCAACGG
MA1776.1	MA1776.1.WRKY53	10.938761	0.952070215	*UFGT*	1965	1973	GGTCAACTT

MA1776.1	MA1776.1.WRKY53	6.072742	0.832534171	*UFGT*	1722	1730	TGTCAAGGA
MA1776.1	MA1776.1.WRKY53	6.036372	0.83164073	*UFGT*	235	243	TGTCAATTA
MA1776.1	MA1776.1.WRKY53	4.9089284	0.803944542	*UFGT*	1939	1947	AGTGAACCC
MA0972.1	MA0972.1.MYBG	15.259513	1.000000007	*CHS*	705	712	AAATATCT
MA0972.1	MA0972.1.MYBG	15.259513	1.000000007	*CHS*	1061	1068	AAATATCT
MA0972.1	MA0972.1.MYBG	12.103007	0.940613216	*CHS*	107	114	AAATATCC
MA0972.1	MA0972.1.MYBG	10.32829	0.907223519	*CHS*	1125	1132	AAATATCA
MA0972.1	MA0972.1.MYBG	8.868829	0.879765079	*CHS*	269	276	AAATATTT
MA0972.1	MA0972.1.MYBG	8.868829	0.879765079	*CHS*	269	276	AAATATTT
MA0972.1	MA0972.1.MYBG	7.607366	0.856031802	*CHS*	1976	1983	AAAAATCC
MA0972.1	MA0972.1.MYB6	8.602051	0.874745893	*DFR*	581	588	AAATTTCT
MA0972.1	MA0972.1.MYB6	5.712323	0.820378288	*DFR*	1407	1414	AAATATTC
MA0972.1	MA0972.1.MYB6	5.690172	0.819961537	*DFR*	1916	1923	TAATACCT
MA0972.1	MA0972.1.MYB6	5.0562963	0.808035738	*DFR*	500	507	TAATCTCT
MA0972.1	MA0972.1.MYB6	5.04674	0.807855945	*DFR*	889	896	GAAAATCT
MA0972.1	MA0972.1.MYB6	4.941379	0.805873673	*DFR*	995	1002	TAATATTT
MA0972.1	MA0972.1.MYB6	10.829601	0.916655236	*ANS*	439	446	AGATATCT
MA0972.1	MA0972.1.MYB6	8.2781105	0.86865125	*ANS*	1907	1914	AAATATGT
MA0972.1	MA0972.1.MYB6	5.0577397	0.808062894	*ANS*	1741	1748	TAATATAT

MA0972.1	MA0972.1.MYB6	10.763871	0.915418584	*UFGT*	522	529	AAAAATCT
MA0972.1	MA0972.1.MYB6	8.985189	0.881954299	*UFGT*	272	279	AAATATAT
MA0972.1	MA0972.1.MYB6	8.868829	0.879765079	*UFGT*	757	764	AAATATTT
MA0972.1	MA0972.1.MYB6	8.534722	0.873479169	*UFGT*	578	585	CAATATCT
MA1038.1	MA1038.1.MYB3	7.7569394	0.84354891	*CHS*	1819	1829	TGGTAGTTAGT
MA1038.1	MA1038.1.MYB3	7.675717	0.841864858	*CHS*	618	628	AAGTTGGTAGC
MA1038.1	MA1038.1.MYB3	6.6303916	0.820191296	*CHS*	1362	1372	GGGGAGGTGGA
MA1038.1	MA1038.1.MYB3	9.59728	0.881706148	*DFR*	1867	1877	ATGTAGGTGGT
MA1038.1	MA1038.1.MYB3	7.542061	0.839093661	*ANS*	1418	1428	TGTTTGGTGGC
MA1038.1	MA1038.1.MYB3	7.266314	0.833376383	*ANS*	322	332	AGTTTGGTGTA

Table S5

Prediction of interaction between TFs

source TF	target TF	score
bHLH137	MYBG	0.546
bHLH137	HY5	0.562
MYB113	HY5	0.964
WRKY53	HY5	0.588
MYB3	HY5	0.798
MYB94	HY5	0.542

Table S6

All primers used in this study

Primer name	Forward 5'-3'	Reverse 5'-3'
y1h-12g885AD	GCCTCTCCCGAATTCATGGAAAAAGACTTCATATCTTG	CCAAAGCTTCTCGAGTCAAAGAGTATCAAGTCCTAGAGAC
y1h-03g1619AD	GCCTCTCCCGAATTCATGGAAAGTGGAAATAATAAGCC	CCAAAGCTTCTCGAGCTAGAGGCTAGCTATAGTCAGGTTTA
y1h-03g1546AD	GCCTCTCCCGAATTCATGGTAGCAGATGCACTGAATCA	CCAAAGCTTCTCGAGTTATGAACACACACGAACCCG
y1h-01g4472AD	GCCTCTCCCGAATTCATGAGAAAATTGCAGCCCA	CCAAAGCTTCTCGAGTCATGAGAAAAAATTTGGATTGT
y1h-10g1791AD	GCCTCTCCCGAATTCATGGAAGTCAATGAAGCAGTAA	CCAAAGCTTCTCGAGCTATAATTTCTCTTTCATAGCTGAA
y1h-08g1044AD	GCCTCTCCCGAATTCATGGAGAAAGTTAAAGGATTGGAGA	CCAAAGCTTCTCGAGTTAACTAAAGAACTCTTCAATGTCA
y1h-10g1433AD	GCCTCTCCCGAATTCATGAATACTGCTATTATTGCCA	CCAAAGCTTCTCGAGCTAATTAAGTAGATTCCATAGGTCA
y1h-DFR-PLACZI	ATTGGATCGGAATTCATAATCACTGCAGGATCTGCATACT	AGCACATGCCTCGAGGAGCCATAGATCCTAAGCTAAATTT
y1h-ANS-PLACZI	ATTGGATCGGAATTCGATACATGTGTAATGCTTTACATTATAT	AGCACATGCCTCGAGCTTAAGCTAAGTCCCCAACACT
y2h-HY5-BD	ATGGAGGCCGAATTCATGCAAGAGCAAGCGACAAGT	CAGGTCGACGGATCCCTACAAGTTGCTGGCTCGAGAC
y2h-MYB113-AD	GAGGCCAGTGAATTCATGAATACTGCTATTATTGCCA	GAGCTCGATGGATCCCTAATTAAGTAGATTCCATAGGTCA
y2h-BHLH143-BD	ATGGAGGCCGAATTC ATGGAAAAAGACTTCATATCTTG	CAGGTCGACGGATCCTCAAAGAGTATCAAGTCCTAGAGAC
VIGS-PDS	CTAGTCTAGAGTCAACTTGAGAGTCCAAGGTA	CGCGGATCCAACTGTATTGTCTAGCTCTGGC
VIGS-MYB113	CCGGAATTCAAGGCCGCATATAAAGAGAGGTGAC	CGCGGATCCATCTATAGTCGACGACGTTTCACTT
RT03g1619	GCACTACTACATCACAAGCAGC	AGTCAGGTTTAATGGATCTCCCTG
RT10g1791	AACCCTGCATGGGTCAGAAA	ACTTTCCCCTGATGTACCTGC
RT05g2190	TGTGGTCAAGGGAGTACGGA	ACATACAGTGTGCCAGAGCC
RT08g1044	CACCAAACCATGACGCAGAA	TGGGACGTTAACAATGGCAC
RT04g1985	GCATCTAGGGAACTTTCATTGGC	CCAGCTAGCAGTAATGCAGGA
RT03g1546	TACTCAGGAGAAGGCACCGA	AAGCACGGCCATACAGCTT
RT01g4472	GGCAACGAAACAAGTGCAGA	ATTCTCGTTTCTCTGGCGGT
RT08g2542	CAGTGGGAGCTGGAACATCA	AGCGCCATTTCGCTAAGATT
RT12g885	CGTGGCTTGATGAGATGGGT	GTTGCAGTATCCACGGGTGA
RT10g1433	CGGGAAGAACAGCAAACGATG	TGCACTTGATGAGAAGGTCCG
RT01g4167	GTGAAGGTACAGGATGGAGAGC	AGGCGTAAAGGTCCCTCTCT
RT03g766	CCGGCCTGGAATTAAACGTG	GTTGTCCGTCCTTTGGGGAA
RT02g906	AAGCAAAGGTGGAATGGGGT	ACTTTAGTAGCTTCAGCCTGGG
RT-CHS	GCCGCACTCATTGTAGGTTC	GGTGACCATCTATAGCGCCT
RT-CHI	ATGGCGTCCATTACTAAAGTGC	TCTAATCCCTGCACCAGCAA
RT-F3H	AGGCAGTAATGGATGAGCCC	CTCAATGGGCATGGATTCCAAC
RT-F3'5'H	GGCCTACAATGCCCAAGACA	ATATCCGCCACCACAACGC
RT-DFR	CGGCTGGATTTATCGGCTCT	CTTCCACGGTCAAGTCTGCT
RT-ANS	TTCTCCTCCCAGACACCGAT	AATCACTCTGTGCTCCACGC
RT-UFGT	AAACAAGGCAATGACACCCC	TTCCTCCTCTGCCTCTTTCA

Figure S1

qRT-PCR Analysis of TFs and light response factors from transcriptome data

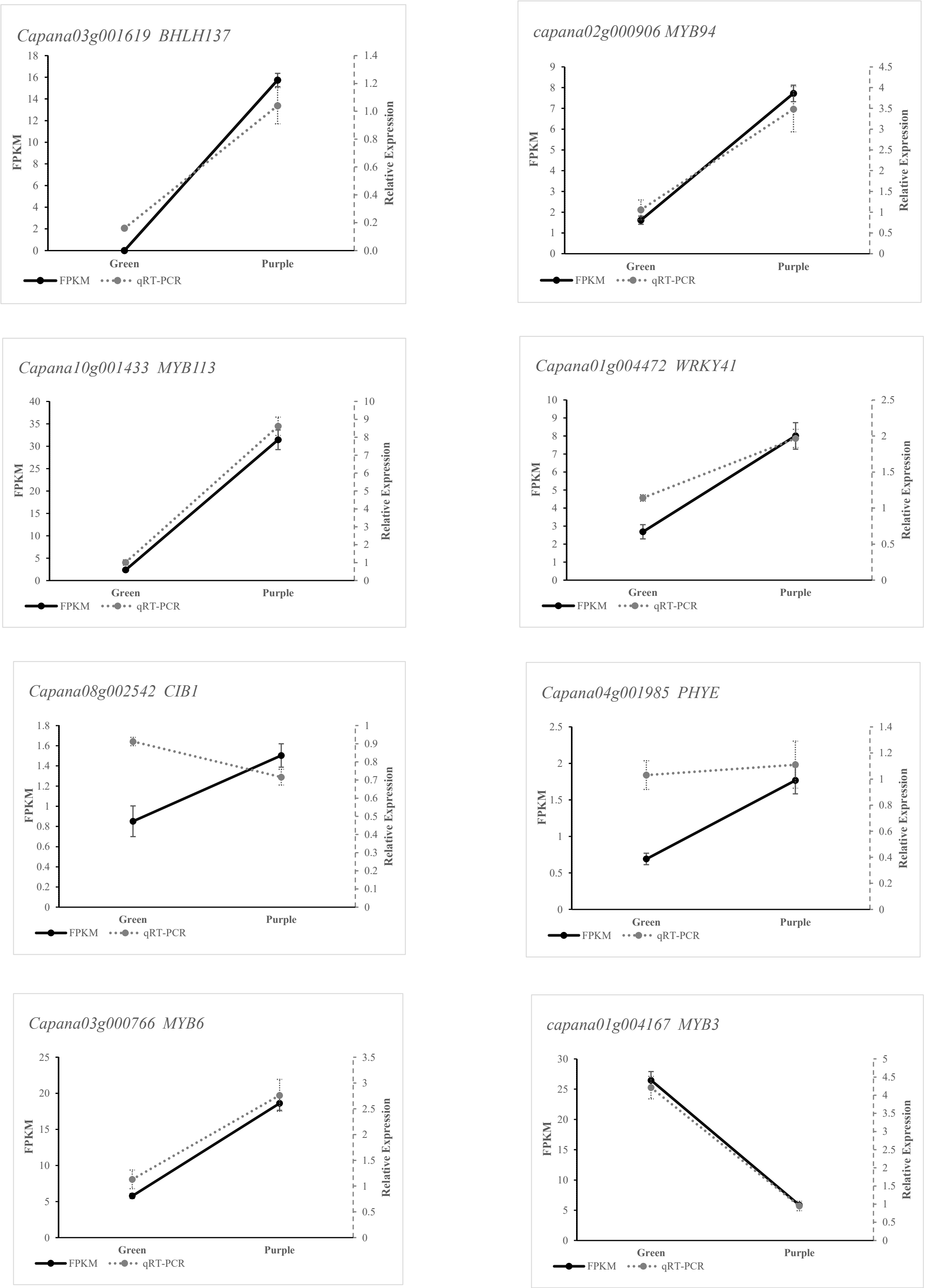

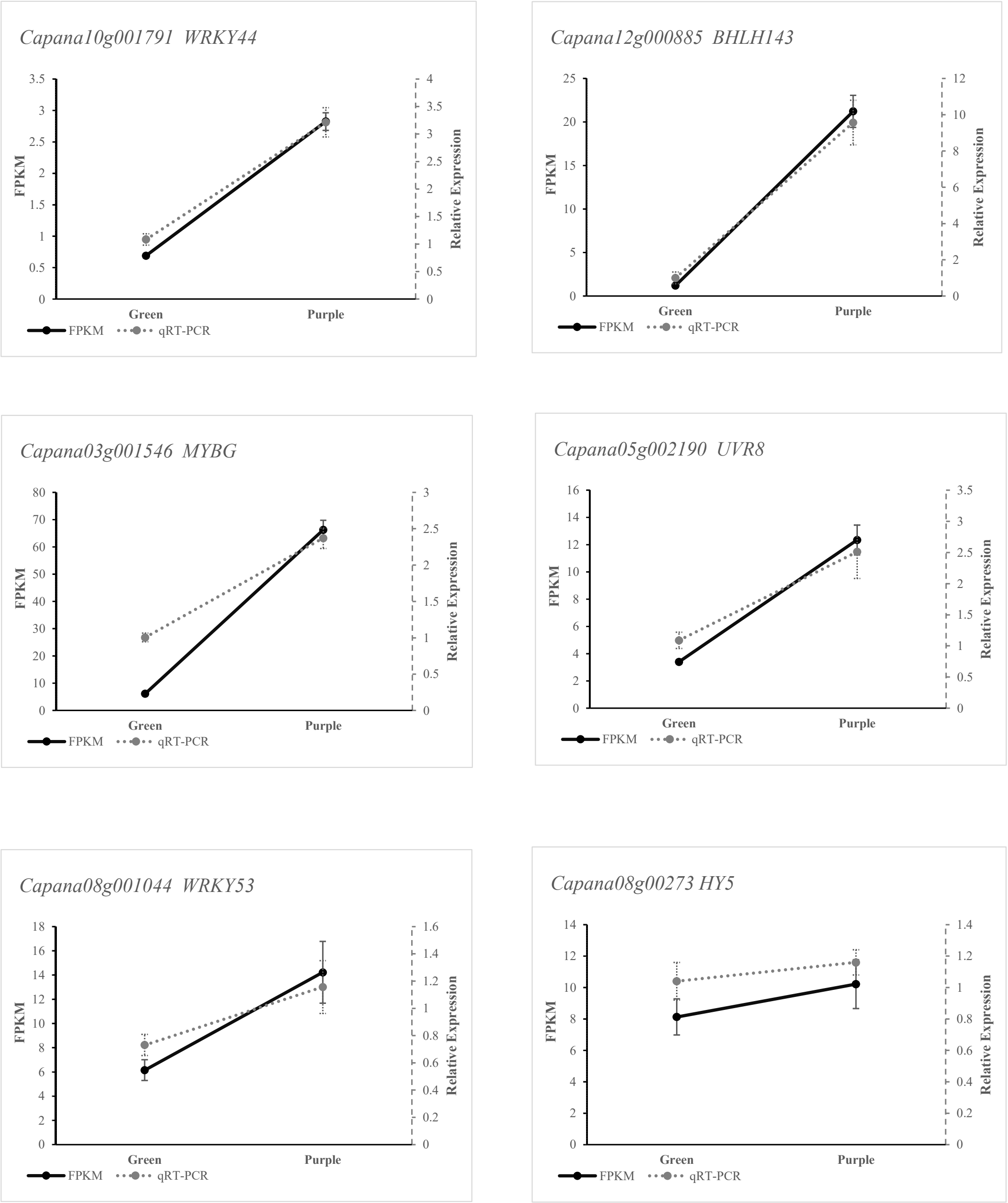

Capana10g001791 WRKY44
FPKM
Relative Expression
Green
Purple
FPKM
qRT-PCR
Capana12g000885 BHLH143
FPKM
Relative Expression
Green
Purple
FPKM
qRT-PCR
Capana03g001546 MYBG
FPKM
Relative Expression
Green
Purple
FPKM
qRT-PCR
Capana05g002190 UVR8
FPKM
Relative Expression
Green
Purple
FPKM
qRT-PCR
Capana08g001044 WRKY53
FPKM
Relative Expression
Green
Purple
FPKM
qRT-PCR
Capana08g00273 HY5
FPKM
Relative Expression
Green
Purple
FPKM
qRT-PCR
